# Measuring the value of the *WHO Model list of essential medicines*

**DOI:** 10.2471/BLT.24.292521

**Published:** 2024-10-01

**Authors:** Elizabeth F Peacocke, Elina Dale, Amani Thomas Mori, Augustina Koduah, Unni Gopinathan

**Affiliations:** a Public Administration and Governance, Faculty of Social Sciences, Oslo Metropolitan University, Pilestredet 35, 0166 Oslo, Norway.; b Global Health Cluster, Norwegian Institute of Public Health, Oslo, Norway.; c Department of Global Public Health and Primary Care, University of Bergen, Bergen, Norway.; d Department of Pharmacy Practice and Clinical Pharmacy, University of Ghana, Accra, Ghana.; e Centre for Epidemic Interventions Research, Norwegian Institute of Public Health, Oslo, Norway.

The *World Health Organization Model list of essential medicines* has guided national medicine selection processes for almost 50 years. This list defines which medicines are more important than others, with the goal of prioritizing and improving their access at an affordable price.

An article in the *Bulletin of the World Health Organization* describes 10 visions to improve the model list.^1^ Proposals focus on the World Health Organization (WHO) Expert Committee process, stressing the importance of involving national stakeholders in decision-making. The authors highlight the need to recognize that not all medicines are equal, calling for more consideration of affordability and cost.^1^ Here we focus on how the model list can support national lists to cover gaps in access to medicines for nationally determined priority conditions.

We offer our views on two areas to enhance the influence of WHO’s model list on access to essential medicines. First, on how the impact of the model list can be more systematically measured and documented. Second, on the need to strengthen the link between WHO model list processes, national lists and key national processes related to financing, reimbursement and procurement of medicines.^2^

More robust measures are needed to assess the impact of the model list, rather than using assessments that may give a distorted picture. Studies of the country-level impact of the model list that compare it with national lists have found varying discrepancies.^3^^–^^5^ Some are justified by the local epidemiological context, while others are less justified.^1^^,^^3^^,^^4^ An example of discrepancy is when the inclusion of medicines on national lists are out of step with evidence-based guidelines.^5^ Questions arise about the risk of wasting resources on ineffective drugs or overlooking alternative drugs with better profiles, especially when national lists include drugs not on the WHO model list.^3^^,^^4^ For example, a study questioned why diclofenac, a non-steroidal anti-inflammatory drug, remained on many countries’ national lists despite being excluded from the WHO model list due to safety concerns, highlighting an unjustified disconnect between the WHO model list and national lists.^5^

These comparisons do not reveal whether inclusion in the WHO model list has mobilized downstream policy tools, including pharmaceutical pricing and procurement policies, that support access to medicines.^6^ Future evaluations of the model list’s impact should quantify whether inclusion in the model list leads to implementation of policy instruments for achieving greater affordability of costly drugs.^6^

Country experiences suggest that access to medicines is sustained through reimbursement lists that are tightly linked to national health policy and financing of pharmaceuticals.^7^^,^^8^ A review of the country-level implementation of the WHO model list found misalignment between national list processes, and processes for determining benefit packages, reimbursement, procurement and clinical guidelines.^9^ To maximize its impact, country-level use of the WHO model list should be integrated with these related processes. In Ghana, for example, the national health insurance benefit package has historically been aligned with the national medicine list and standard treatment guidelines.^10^ However, since the last list was updated in 2017, new medicines have been added to Ghana’s benefit package that are not on the list. This finding highlights the challenge of keeping both the WHO model list and national medicine lists relevant for key decisions that influence access to medicines nationally. 

Promoting such a link does not imply that every inclusion on the WHO model list should automatically translate into inclusion in a country’s health benefit package. However, countries striving for universal health coverage could leverage evidence generated during the WHO model list process on effectiveness and safety to fast-track decision-making processes and enable access to medicines. In Ukraine, the WHO model list is used to prequalify medicines for the national medicine list, so long as there is sufficient domestic competition of international non-proprietary named medicines.^11^ This approach is an innovative solution for countries with evolving domestic capacity for health technology assessments.

In conclusion, the WHO model list can have a greater impact on access to medicines if it better supports decisions about outpatient drug benefit packages, which are key for lowering out-of-pocket payments.^12^ The model list should also promote better linkages to the implementation of policy tools that shape the broader medicines ecosystem, including pharmaceutical pricing and procurement policies. 
